# Diversification into novel habitats in the Africa clade of *Dioscorea* (Dioscoreaceae): erect habit and elephant’s foot tubers

**DOI:** 10.1186/s12862-016-0812-z

**Published:** 2016-11-08

**Authors:** Olivier Maurin, A. Muthama Muasya, Pilar Catalan, Eugene Z. Shongwe, Juan Viruel, Paul Wilkin, Michelle van der Bank

**Affiliations:** 1 African Centre for DNA Barcoding, Department of Botany & Plant Biotechnology, University of Johannesburg, PO Box 524, Auckland Park, Johannesburg, Gauteng 2006 South Africa; 2 Royal Botanic Gardens, Kew, Richmond, Surrey, TW9 3AE UK; 3 Department of Biological Sciences, University of Cape Town, Rondebosch, Western Cape 7701 South Africa; 4 Departamento de Ciencias Agrarias y del Medio Natural, Escuela Politécnica Superior de Huesca, Universidad de Zaragoza, Ctra. Cuarte km 1, Huesca, 22071 Spain; 5 Institute of Biology, Tomsk State University, 36 Lenin Avenue, Tomsk, 634050 Russia; 6 Departamento de Biología Vegetal y Ecología, Facultad de Biología, Universidad de Sevilla, Av. Reina Mercedes s/n, Sevilla, 41012 Spain; 7 Institut Méditerranéen de Biodiversité et d’Ecologie marine et continentale (IMBE), Aix Marseille Université, Chemin de la Batterie des Lions, Marseille, 13007 France

**Keywords:** Biogeography, Dioscoreales, “elephant’s foot”, Fire adaptation, Habitat transition, Pachycaul, Southern Africa, Yams

## Abstract

**Background:**

*Dioscorea* is a widely distributed and highly diversified genus in tropical regions where it is represented by ten main clades, one of which diversified exclusively in Africa. In southern Africa it is characterised by a distinct group of species with a pachycaul or “elephant’s foot” structure that is partially to fully exposed above the substrate. In contrast to African representatives of the genus from other clades, occurring mainly in forest or woodland, the pachycaul taxa and their southern African relatives occur in diverse habitats ranging from woodland to open vegetation. Here we investigate patterns of diversification in the African clade, time of transition from forest to more open habitat, and morphological traits associated with each habitat and evaluate if such transitions have led to modification of reproductive organs and mode of dispersal.

**Results:**

The Africa clade originated in the Oligocene and comprises four subclades. The *Dioscorea buchananii* subclade (southeastern tropical Africa and South Africa) is sister to the East African subclade, which is respectively sister to the recently evolved sister South African (e. g., Cape and Pachycaul) subclades. The Cape and Pachycaul subclades diversified in the east of the Cape Peninsula in the mid Miocene, in an area with complex geomorphology and climate, where the fynbos, thicket, succulent karoo and forest biomes meet.

**Conclusions:**

Diversification out of forest is associated with major shifts in morphology of the perennial tuber (specifically an increase in size and orientation which presumably led them to become pachycaul) and rotation of stem (from twining to non-twining). The iconic elephant's foot morphology, observed in grasslands and thicket biomes, where its corky bark may offer protection against fire and herbivory, evolved since mid Miocene. A shift in pollination trait is observed within the forest, but entry into open habitat does not show association with reproductive morphology, except in the seed wing, which has switched from winged all round the seed margin to just at the base or at the apex of it, or has been even replaced by an elaiosome.

**Electronic supplementary material:**

The online version of this article (doi:10.1186/s12862-016-0812-z) contains supplementary material, which is available to authorized users.

## Background

*Dioscorea* L. is a monocotyledonous plant genus that is highly diverse in many tropical regions of the world, with comparatively few taxa found in temperate latitudes. It comprises over 600 species, almost all of which have perennating organs (rhizome and/or tuber). These organs give rise to herbaceous, usually twining stems bearing leaves with basal and apical petiolar pulvinii and campylodromous venation. Most species are dioecious, with relatively small, typically monocotyledonous trimerous flowers in spicate or racemose (partial) inflorescences, with female plants usually containing up to six (usually) winged seeds in each inferior ovary. The highest species diversity per unit area is found in tropical areas, for example, southern Brazil, parts of Mexico, the Greater Antilles, western Madagascar and Asia from southern China to the Isthmus of Kra in Thailand [1–5]. These are largely areas with seasonal climates supporting open, deciduous forests that allow these light-demanding plants to thrive.

Wilkin et al. [6] established the broad phylogenetic outline of *Dioscorea*, which comprises 10 main clades. The same tree topology has been supported through significantly increased sampling and a further plastid marker [7] as well as was with additional data from the nuclear region *Xdh*, (Viruel, personal communication). The first branching group, the Stenophora clade (Fig. 3), is rhizomatous, with its highest diversity in subtropical Asia, followed by two large clades endemic to the Neotropics. The remaining clades comprise smaller units of diversity from the Mediterranean and Africa plus the principal reservoirs of species numbers in the Caribbean, Madagascar and the palaeotropics as a whole. Thus the focus of research in this genus has now shifted to species forming these 10 major clades.

Of those 10 major clades, three are distributed in sub-Saharan Africa. One of these is the first *Dioscorea* clade to be studied here via a species level phylogeny, the Africa clade of Viruel et al. [7]. It is also the only clade to have diversified exclusively in Africa and comprises 13 species, as listed in [1] with minor taxonomic changes made in [8]. Nine are South African (sub)endemic species, two extend from South Africa into southern tropical Africa (*D. buchananii* Benth. and *D. sylvatica* Ecklon) and two are disjunct in northeastern tropical Africa (*D. gillettii* Milne-Redh. and *D. kituiensis* Wilkin & Muasya). In contrast, the remaining *Dioscorea* clades found in sub-Saharan Africa sensu [7] are poorly represented in South Africa, with only one species in the Enantiophyllum clade (*D. cotinifolia* Kunth; Fig. 3) and three in the Compound Leaved clade (CL; Fig. 3). This contrasts with the substantial tropical African diversity in these lineages.

**Fig. 1:**
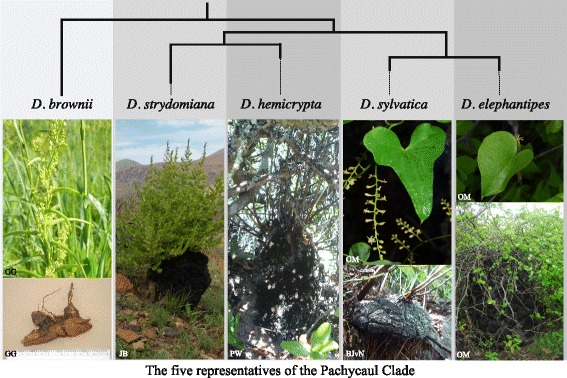
Mapping of habit, tuber and leaf traits on five South Africa yam lineages of the Pachycaul clade. Photographs: BJvN = Brian J van Niekerk; GG = Graham Grieve; JB = John Burrows; OM = Olivier Maurin; PW = Paul Wilkin

The species of the Africa clade (Fig. 3) of Viruel et al. [7] possess a number of distinctive or unusual morphological traits. They include perennial tubers, some of which are large, “elephant’s foot” pachycaul structures that are partially or wholly exposed from the substrate (Fig. 1; [9, 10]). Similar structures also occur infrequently in neotropical species such as *D. mexicana* Scheidw.; two of the main neotropical lineages of *Dioscorea* also possess perennial tubers. Stems are usually sinistrorse (climbing towards the left hand, as viewed externally) but in some taxa they are non-twining [10, 11]. This trait is also encountered elsewhere, for example in the Mediterranean clade (*D. pyrenaica* Bubani & Bordère ex Gren. and *D. chouardii* Gaussen; Fig. 3) from Pyrenean France and Spain, in the *Epipetrum* group from Chile [12], and *D. hexagona* Baker from Madagascar [13]. Leaves are always alternate and blades are entire to deeply palmately lobed. Stamen number is reduced from 6 to 3 in one species [14]. Seeds in the Africa clade vary from possessing a wing all round the margin of the seed with a longer and shorter axis to being winged just at the apex [10, 15]. This is correlated with a capsular fruit that is longer than broad. *Dioscorea gillettii* and *D. kituiensis* have seeds that are wingless but possess an aril-like structure [16].

Among *Dioscorea* species occurring in Africa, the Africa clade is the richest source of steroidal saponins [17, 18]. *Dioscorea sylvatica* in particular was extracted from the wild in South Africa in the 1950s to produce synthetic human hormones for contraceptive purposes and other steroidal drugs. In contrast, some taxa of the CL clade have alkaloid chemistry [18] which is the basis of the use of *D. dregeana* in South African traditional medicine (e.g. [19]). The principal use of Enantiophyllum clade species is as a starch source that feeds at least 60 million people in tropical Africa [20].

The species of the CL and the Enantiophyllum clades are typical of the genus as a whole in that they mainly inhabit forest or woodland biomes, often those that are seasonal in climate. However, the Africa clade occupies an unusually broad range of vegetation types for the genus, including not only afromontane forests or forest margins and savannah woodlands but also the fynbos heathlands, succulent karoo and thicket. This observation reinforces three key questions that this research sets out to investigate. First, what are the patterns and timing of diversification in the Africa clade, especially in relation to transitions from forest to more open habitats such as thickets and karoo? Second, how are the traits associated with forest or woodland habitats modified in taxa inhabiting more open biomes, especially vegetative traits of perennating organs, stems and leaves, including their size and shape? Finally, are floral and fruit reproductive traits similarly affected by these biome shifts in addition to vegetative traits?

## Methods

### Taxon sampling

Representatives of all known African perennial-tubered *Dioscorea* (Dioscoreaceae) were sampled (Table 1). These included five pachycaul species, three Cape species, three species of the *D. buchananii* subclade (as defined by Wilkin and Muasya [8]), two species of the southern African members of the CL clade, and two species from the Enantiophyllum clade as well as two species from the East Africa subclade. We also included representatives from all known *Dioscorea* lineages [6]: four from the Mediterranean clade, *D. tentaculigera* and *D. prazeri* from South-East Asia and two New World taxa (*D. brachybotrya* and *D. galeottiana*), respectively belonging to the New World I (NWI) and II clades (NWII); and *Tacca* and *Stenomeris* (Dioscoreaceae) were selected as outgroups. Voucher specimen information and GenBank accession numbers are listed in Table 1 and trace files and sequences are available on the Barcode of Life Data System (BOLD; www.boldsystems.org).

**Table 1:** Table of material and Genbank accession. Collectors references acronyms are Olivier Maurin (OM), Muthama Muasya (AMM), Sebsebe Demissew (SD), Pilar Catalán (PC), Ernesto Pérez-Collazos (EP). Genbank accessions in plain text are new to this study, accessions in bold were retrieved ﻿from www.ncbi.nlm.nih.gov

Taxon	Voucher *(Museum)*	Distribution	Habitat	Habit	rbcL	matK	trnL-F	trnH-psbA	PsaA-Ycf3	rpl32-trnL
*Dioscorea brachybotrya* Poepp.	Rudall P. s.n *(K)*	C. & S. Chile to Argentina	At medium altitude up to the timber line, and at low altitude in interior valleys	Shrub climber, 2 m in height	**AF307469**	**AY956482**	**KM878027**	KR086979	KR087124	KR088291
*Dioscorea brownii* Schinz	Grieve 53 *(K)*	South Africa - KwaZulu-Natal	Grassland	Pachycaul slightly emerging with annual solitary stems reaching 1 m in height	KR087028	KR086942	KR070855	KR086980	KR087125	KR088292
*Dioscorea buchananii* Benth.	Bingham10290 *(K)*	Tanzania, Angola, Malawi, Mozambique, Zambia, Zimbabwe, DRC	*Combretum* thickets and at proximity of woody outcrop	Twining wine with perennial tuber and stems up to 9 m long	KR087029	KR086943	KR070856	KR086981	KR087126	KR088293
*Dioscorea bulbifera* L.	OM3576 *(BNRH)*	Native in Tropical and subtropical region from Africa, Asia and Australasia. Introduced elsewhere	Forest and woodlands	Twining wine with perennial tuber and stems up to 12 m long	KR087030	KR086944	KR070857	KR086982	KR087127	KR088294
*Dioscorea burchellii* Baker	AMM6650 *(BOL)*	South Africa - Eastern Cape	At medium to high Altitude in dense fynbos vegetation	Tuberous perennial, shoot to 1 m height	KR087032	KR086946	KR070859	KR086984	KR087129	KR088296
*Dioscorea burchellii* Baker	AMM6704A *(BOL)*	-	-	-	KR087031	KR086945	KR070858	KR086983	KR087128	KR088295
*Dioscorea chouardii* Gaussen	PC334 *(JACA)*	Spain	Limestone rock-crevices	Tuberous perennial, shoot to 1 m height	**KM877855**	**KM877907**	KR070860	KR086985	KR087130	KR088297
*Dioscorea communis* (L.) Caddick & Wilkin	Chase536 *(K)*	Europe, North African and temperate Asia	Woodland and woodland hedges	Herbaceous with climbing stem, up to 4 m in height	KR087033	KR086947	KR070871	KR086996	KR087141	KR088308
*Dioscorea cotinifolia* Kunth	AMM6112 *(BOL)*	Mozambique, South Africa and Swaziland	Open dry forest, forest margins, scrubby vegetation and rocky places	Tuberous plant with vigorous annual twining stems reaching up to 10 m height	KR087034	KR086948	KR070862	KR086987	KR087132	KR088299
*Dioscorea cotinifolia* Kunth	AMM6158 *(BOL)*	-	-	-	KR087035	KR086949	KR070863	KR086988	KR087133	KR088300
*Dioscorea cotinifolia* Kunth	OM1458 *(BNRH)*	-	-	-	KR087036	KR086950	KR070864	KR086989	KR087134	KR088301
*Dioscorea dregeana* (Kunth) T. Durand & Schinz	AMM6104 *(BOL)*	Mozambique and South Africa	Forest, woodlands found among rocks and ravine	Tuberous plants with annual to persistent stems reaching up to 12 m height	KR087038	KR086952	KR070866	KR086991	KR087136	KR088303
*Dioscorea dregeana* (Kunth) T. Durand & Schinz	AMM6166 *(BOL)*	-	-	-	KR087039	KR086953	KR070868	KR086993	KR087138	KR088305
*Dioscorea dregeana* (Kunth) T. Durand & Schinz	OM1465 *(BNRH)*	-	-	-	JQ025042	JQ024957	KR070867	KR086992	KR087137	KR088304
*Dioscorea dregeana* (Kunth) T. Durand & Schinz	OM2247 *(BNRH)*	-	-	-	KR087037	KR086951	KR070865	KR086990	KR087135	KR088302
*Dioscorea dumetorum* (Kunth) Pax	OM2315 *(BNRH)*	Sub-Saharan Africa, excluding parts of Southern Africa	Forest and woodlands and along riverbanks, generally at low altitude	Tuberous plants with annual to persistent stems reaching up to 5 m height	KR087040	KR086954	KR070869	KR086994	KR087139	KR088306
*Dioscorea dumetorum* (Kunth) Pax	OM3953 *(BNRH)*	-	-	-	KR087041	KR086955	KR070870	KR086995	KR087140	KR088307
*Dioscorea elephantipes* (L’Hér.) Engl.	AMM5225 *(BOL)*	Namibia, South Africa - All Capes provinces	From Medium to high altitude, in thorny and succulent vegetation (e.g. Thickets)	Pachycaul up to 80 cm in diameter, with shoot up to 1 m height	KR087042	KR086956	KR070872	KR086997	KR087142	KR088309
*Dioscorea elephantipes* (L’Hér.) Engl.	AMM5226a *(BOL)*	-	-	-	KR087043	KR086957	KR070873	KR086998	KR087143	KR088310
*Dioscorea elephantipes* (L’Hér.) Engl.	AMM6713 *(BOL)*	-	-	-	KR087044	KR086958	KR070874	KR086999	KR087144	KR088311
*Dioscorea galeottiana* Kunth	Telez13090 *(MEXU)*	Centre America, Mexico	Tropical Dry Forest	Perennial tuber, climber	**AY904796**	**AY956499**	**KM878046**	KR087000	KR087145	KR088312
*Dioscorea gillettii* Milne-Redh.	SD7051 *(ETH)*	Southern Ethiopia	Dry woodland vegetation	Perennial tuber with twining stems up to 1.5 m height	KR087046	KR086960	KR070876	KR087002	KR087147	KR088314
*Dioscorea gillettii* Milne-Redh.	SD7052 *(ETH)*	-	-	-	KR087045	KR086959	KR070875	KR087001	KR087146	KR088313
*Dioscorea hemicrypta* Burkill	AMM5800 *(BOL)*	South Africa - Western Cape	From Medium to high altitude, in thorny and succulent vegetation (e.g. Thickets)	Pachycaul, partially emerged with annual shoot emerging from the crown	KR087050	KR086964	KR070880	KR087006	KR087151	KR088318
*Dioscorea hemicrypta* Burkill	AMM6633 *(BOL)*	-	-	-	KR087049	KR086963	KR070879	KR087005	KR087150	KR088317
*Dioscorea hemicrypta* Burkill	AMM6886a *(BOL)*	-	-	-	KR087047	KR086961	KR070877	KR087003	KR087148	KR088315
*Dioscorea hemicrypta* Burkill	AMM6697 *(BOL)*	-	-	-	KR087048	KR086962	KR070878	KR087004	KR087149	KR088316
*Dioscorea kituiensis* Wilkin & Muasya	Mwachala 949a *(K)*	Eastern Kenya	Rocky area in dry woodlands	Tuberous perennial with twining stems reaching 1.5 m height	KR087051	KR086965	KR070881	KR087007	KR087152	KR088319
*Dioscorea multiloba* Kunth	AMM6167 *(BOL)*	South Africa - Eastern Cape, KwaZulu-Natal and Swaziland	High altitude forests vegetation.	Tuberous perennial with twining stems reaching 2 m in height	KR087052	KR086966	KR070882	KR087008	KR087153	KR088320
*Dioscorea mundii* Baker	AMM6641 *(BOL)*	South Africa - Western Cape	Coastal forest vegetation	Perennial with underground tuber from which stems arise (or from the bases of old stems) that climb to at least 5 m in height in surrounding vegetation	KR087054	KR086968	KR070884	KR087010	KR087155	KR088322
*Dioscorea mundii* Baker	AMM6642 *(BOL)*	-	-	-	KR087053	KR086967	KR070883	KR087009	KR087154	KR088321
*Dioscorea orientalis* (J. Thiébaut) Caddick & Wilkin	Tori 1 *(HUJ)*	Temperate western Asia	Mediterranean Woodlands and Shrublands	Geophyte, climber	**KM877858**	**KM877911**	**KM878066**	KR087011	KR087156	KR088323
*Dioscorea prazeri* Prain & Burkill	Wilkin1075 *(K)*	Asia	Open vegetation in mixed forests	Rhizome, with climbing stem up to 5 m height	**AY973485**	**KM877871**	**KM878019**	KR087012	KR087157	KR088324
*Dioscorea pyrenaica* Bubani & Bordère ex Gren.	EP1038 *(JACA)*	France, Spain (Pyrenees mountain range)	On limestone rocks	Tuberculous plant with short annual stems reaching 40 cm height	**KM877859**	**KM877912**	**KM878067**	KR087013	KR087158	n.a.
*Dioscorea rupicola* Kunth	AMM3676 *(BOL)*	South Africa - Eastern Cape, KwaZulu-Natal	Occurring in open areas at high altitude in shady temperate and humid forest	Perennial tuber with twining stems growing on surrounding vegetation	KR087055	KR086969	KR070885	KR087014	KR087159	KR088325
*Dioscorea sansibarensis* Pax	OM2421 *(BNRH)*	Sub-Saharan Africa, including Madagascar but excluding Southern Africa	Humid forest, low altitude, along riverine	Large tuberous species with vigorous stems up to 30 m in length climbing and trailing on surrounding vegetation	KR087056	KR086970	KR070886	KR087015	KR087160	KR088326
*Dioscorea schimperana* Hochst. ex Kunth	OM2372 *(BNRH)*	Tropical Africa, including Zambia, Zimbabwe, Malawi and Mozambique	Open vegetation, on Rocks, termites’ mounts and along riverbanks	Annual tuber, producing vigorous shoots reaching 8 m height	KR087058	KR086972	KR070888	KR087017	KR087162	KR088328
*Dioscorea schimperana* Hochst. ex Kunth	OM3532 *(BNRH)*	-	-	-	KR087057	KR086971	KR070887	KR087016	KR087161	KR088327
*Dioscorea stipulosa* Uline ex R. Knuth	AMM6748 *(BOL)*	South Africa - Eastern Cape	In fynbos, in moist rich soils	Perennial tuber with annual shoot reaching up to 3 m in length	KR087060	KR086974	KR070890	KR087019	KR087164	KR088330
*Dioscorea stipulosa* Uline ex R. Knuth	AMM6800 *(BOL)*	-	-	-	KR087059	KR086973	KR070889	KR087018	KR087163	KR088329
*Dioscorea strydomiana* Wilkin	AMM6124 *(BOL)*	South Africa, Mpumalanga	Open woodland	Pachycaul up to 80 cm in diameter, with shoot up to 90 cm height	KF147467	KF147390	KR070892	KR087021	KR087166	KR088332
*Dioscorea strydomiana* Wilkin	Burrows 10627 *(BNRH)*	-	-	-	KR087061	KR086975	KR070891	KR087020	KR087165	KR088331
*Dioscorea sylvatica* Eckl.	Burrows 12487 *(BNRH)*	Southern Africa	Found from low to high altitude in a variable range of vegetation from Dunes, to rocky outcrop and open woodland vegetation.	Perennial tuber, with Herbaceous annual stem reaching 4 m in length	KR087062	KR086976	KR070893	KR087022	KR087167	KR088333
*Dioscorea sylvatica* Eckl.	OM1433 *(BNRH)*	-	-	-	KR087063	KR086977	KR070894	KR087023	KR087168	KR088334
*Dioscorea sylvatic*a Eckl. f. glauca	Burrows 12477 *(BNRH)*	-	-	-	KR087064	KR086978	KR070895	KR087024	KR087169	KR088335
*Dioscorea tentaculigera* Prain & Burkill	Thapyai 436	South Central Chine, Myanmar and Thailand	Evergreen forest from medium to high altitude	Perennial tuber, with climbing stems up to 4 m in length	**AY972828**	**AY939886**	**KM878070**	KR087025	KR087170	KR088336
*Tamus edulis* Lowe (combination in *Dioscorea* pending)	Chase 3425 *(K)*	Mediterranean region	Woodland and woodland hedges	Herbaceous with climbing stem	**AY939891**	**AY973843**	KR070861	KR086986	KR087131	KR088298
*Outgroups*										
*Stenomeris borneensis* Oliv.	Brun19174 *(K)*	Tropical Asia	-	-	**AF307475**	**AY973836**	-	-	-	-
*Tacca plantaginea* (Hance) Drenth	ZL002 (n.a.)	Tropical Asia	-	-	**JF944619**	**JF956650**	-	-	-	-

### DNA extraction, amplification, sequencing and alignment

DNA was extracted from 0.3 g silica gel dried leaves [21] using 2x CTAB method [22] with the addition of 2 % polyvinyl pyrrolidone (PVP) to reduce the effects of high polysaccharide concentration in the samples. In order to avoid problems of PCR inhibition, DNA was precipitated in 2.5 volume ethanol and purified using QIAquick PCR Purification Kit according to manufacturer’s protocol (QiIAgen Inc., Hilden, Germany). All PCR reactions were carried out using Thermo Scientific Master Mix (Thermo Fischer Scientific, Waltham, Massachusetts, USA).

Amplification of *rbcLa* was carried out using the primers rbcLa-F and rbcLa-R described respectively by Levin et al. [23] and Kress and Erickson [24]. For *matK*, the following primers were used MatK-1R-Kim-F and MatK-3 F-Kim-R (Kim, unpublished; [25]). Amplification of *trnL-F* was carried out using primers c and f of Taberlet et al. [26], but the internal primers d and e were also used for several taxa due to difficulty in amplifying the region as a single piece. The *trnH-psbA* spacer was amplified using primers 1 F and 2R [27]. The *psaA-ycf3* spacer was amplified using the PG1f and PG2r primers [28]. Finally the *rpl32-trnL* ^(UAG)^ intergenic spacer was amplified according to Shaw et al. [29]. Amplified products were purified using QIAquick columns (QIAgen, Germany) following the manufacturer’s protocol.

PCR amplification primers were also used as cycle sequencing primers. Cycle sequencing reactions were carried out using BigDye© V3.1 Terminator Mix (Applied Biosystems, Inc., ABI, Warrington, Cheshire, UK) and cleaned using the EtOH-NaCl method provided by ABI; they were then sequenced on an ABI 3130xl genetic analyser. Complementary strands were assembled and edited using Sequencher version 5.1 (Gene Codes Corp., Ann Arbor, Michigan, USA) and sequences were aligned manually in PAUP* (version 4.0b1; [30]) without difficulty due to low levels of insertions/deletions.

### Phylogenetic analyses: parsimony and Bayesian approaches

Maximum parsimony (MP) using PAUP* version 4.0b1 [30] was performed on the individual and combined datasets. Tree searches were conducted using 1,000 replicates of random taxon addition, retaining 10 trees at each step, with tree-bisection-reconnection (TBR) branch swapping and MulTrees in effect (saving multiple equally parsimonious trees). Support for clades in all analyses was estimated using bootstrap analysis [31] with 1000 replicates, simple sequence addition, TBR swapping, with MulTrees in effect but saving a maximum of 10 trees per replicate. Delayed transformation character optimization (DELTRAN) was used to calculate branch lengths, due to reported errors http://paup.sc.fsu.edu/paupfaq/paupans.html with accelerated transformation optimization (ACCTRAN) in PAUP v.4.0b1. Bootstrap support (BP) was classified as high (85–100 %), moderate (75–84 %) or low (50–74 %). Bootstrap values are provided in Fig. 3. All data sets were analyzed separately, and the individual bootstrap consensus trees examined by eye to identify topological conflicts, i.e. moderate to high support for different placement of taxa. In order to test for significant conflicts between the independent DNA data matrices, a partition homogeneity test was performed [32–34]. The Incongruence Length Difference (ILD) test of Farris et al. [32] implemented in PAUP* 4.0 b10 [30] was performed through 1000 random-order-entry replicates to estimate if the six datasets were significantly different from random partitions of the same size. Non-significant results indicated that the six data sets were not heterogeneous. Highly congruent contrasted topologies (see Results) also supported the merging of the four data matrices into a single concatenated data set that was used for subsequent phylogenetic analyses.

Bayesian analysis (BI; [35, 36]) was performed using MRBAYES v. 3.1.2. For each matrix *rbcLa*, *matK*, *trnL-F*, *trnH-psbA*, *psaA-ycf3* and *rpl32-trnL* the most appropriate model was selected using MODELTEST v. 3.06 [37]. For *matK* and *trnL-F* the model TVM + G was selected, then for *rbcLa*, *trnH-psbA*, *psaA-ycf3* and *rpl32-trnL*, the following model were selected, respectively TVM + I, HKY + G, HKY + I + G and GTR + G. The analysis was run on the CIPRES cluster [38] using a MCMC of 10 million generations with a sample frequency of 500, imposing the closest nst = 6 rates = gamma model available in the program. The resulting trees were plotted against their likelihoods to determine the point where likelihoods converged on a maximum value, and all the trees before the convergence were discarded as ‘burn-in’ (5000 trees). All remaining trees were imported into PAUP 4.0b10, and a majority-rule consensus tree was produced showing frequencies (i.e. posterior probabilities or PP) of all observed bi-partitions. The following scale was used to evaluate the PPs values: below 0.95, weakly supported; 0.95-1.00, well supported.

### Divergence time estimation

Divergence times were estimated using a Bayesian MCMC approach implemented in BEAST (v. 1.4.8; [39]), which allows simultaneous estimation of the topology, substitution rates and node ages [39]. The GTR + I + G implemented model of sequence evolution for each partition based on the Akaike information criterion (AIC) scores for substitution models evaluated using MrModeltest (version 2.3; [40]) with a gamma-distribution with four rate categories. A speciation model following a Yule process was selected as the tree prior, with an uncorrelated lognormal (UCLN) model for rate variation among branches. For this analysis, we used a single representative per species since the Yule speciation model forces the analysis to “create” speciation events at every node and therefore makes the estimation of splits older within a species.

First, the Bayesian consensus tree topology was used as a starting tree and adjusted so that branch lengths satisfied all fossil prior constraints, using PATHd8 v.1.0 [41]. Fossil dates or calibration points were used to constrain specific nodes to minimum, maximum or fixed ages. The crown node age of Dioscoreaceae was calibrated at 80 mya according to Jansen & Bremer [42]. A first fossil, *Dioscorea lyelli* (Wat.) Fritel, was used to calibrate the node of *Dioscorea prazeri* Prain & Burkill*,* (representative of the *Stenophora* clade). The fossil was discovered in the Cuisian stage of the Ypresian age at the Paris basin [43] and provided a minimum constraint of 48.2 ± 1.0 mya (LogNormal Prior mean = 48.2, SD 0.008) for the stem node of *Stenophora*. A second fossil, *D. wilkinii* Pan, attributed to the node of the Compound Leaved clade that comprises *D. dregeana - D. dumetorum,* provided a minimum constraint of 27.2 ± 0.1 mya (LogNormal Prior mean = 27.2, SD 0.002) for that node [44]. We performed four independent runs of MCMC, each for 100 million generations, sampling every 1000 generations. We assessed the MCMC log files for convergence using the effective sample size (ESS) statistics in Tracer v.1.5 [39]. The BEAST analysis reported ESS values > 200, indicating that the posterior estimates were not unduly influenced by autocorrelation. The resulting tree files from the four runs were then combined using LogCombiner v.1.7.5 [39], discarding the first 25 % trees as burn-in. The maximum clade credibility consensus tree, with means and 95 % highest posterior density (HPD) intervals, was generated with TreeAnnotator v.1.7.5 [39].

### Map preparation

**Fig. 2:**
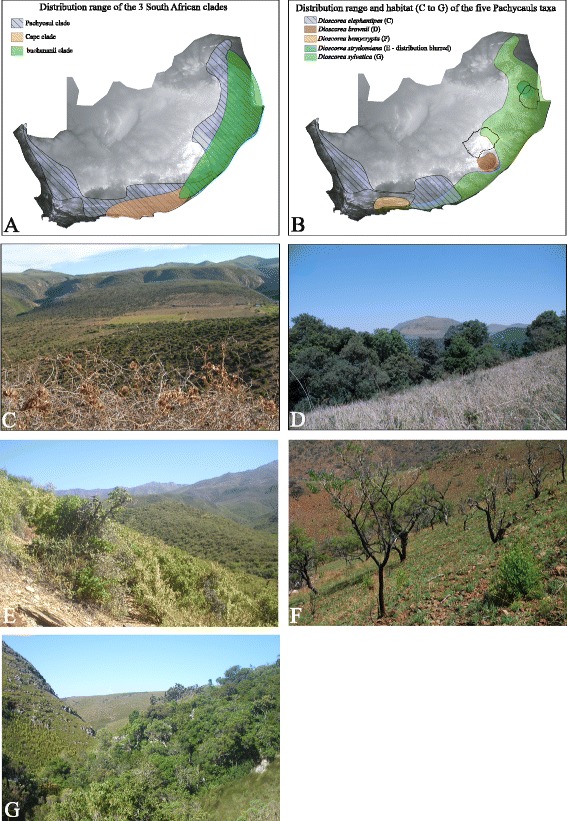
Distribution maps and habitat images of the southern African *Dioscorea* taxa. **a** Distribution map of the three South African subclades of the Africa clade: Pachycaul, Cape and *D. buchananii*. **b** Distribution map of the five Pachycaul subclade species in South Africa. Note that the distribution of *D. elephantipes* extends slightly into Namibia, and *D. sylvatica* extends into Mozambique, Zambia and Zimbabwe. From **c** to **g**: in order, habitat of *D. elephantipes*, *Dioscorea brownii*, *D. hemycrypta*, *D. strydomiana,* and *D. sylvatica*. The habitat image of *D. elephantipes* (**c**) displays in the foreground shoots and fruits of this taxa; the habitat image for *D. strydomiana* has an immature or damaged specimen in the bottom right corner. All other images only show the habitat and individuals of the species are not visible. Photographs: **c-g**: Paul Wilkin

Distribution maps illustrated on Fig. 2 were prepared using occurrence data downloaded from http://newposa.sanbi.org and http://www.gbif.org. Distribution ranges were drawn on Adobe ® Illustrator ® CS6. Figure 2a represents the occurrence of the three major subclades occurring in South Africa, the Pachycaul, Cape and *D. buchananii* subclades, while Fig. 2b displays the distribution of all species belonging to the Pachycaul subclade.

## Results

Statistics for MP analysis for the six plastid markers and combined dataset are presented in Table 2. Of all the genes used, *matK* and *rpl32-trnL* had a significantly higher number of variable sites (27.85 % and 25.17 % respectively) compared to the other regions than display percentages below 10 % (see Table 2). The number of potentially informative characters is higher for *matK* (12.07 %) than *rpl32-trnL* (10.01 %), however contribution to total of parsimony informative character (PIC) is lower for *matK* (26.35 %) than for *rpl32-trnL* (30.84 %; Table 2).

**Table 2:** Maximum parsimony statistics from the analyses of the separate and combined data sets

	*rbcLa*	*matK*	*trnL-F*	*trnH-psbA*	*psaA-ycf3*	*rpl32-trnL*	*Combined*
No. of taxa	49	49	47	47	47	47	49
No. of included characters (= aligned length)	529	729	738	409	709	1029	4143
No. of constant characters	472	526	613	361	602	770	3344
No. of variable sites	57	203	125	48	107	259	799
	(10.77 %)	(27.85 %)	(16.94 %)	(11.74 %)	(15.09 %)	(25.17 %)	(19.29 %)
No. of parsimony informative character (PIC)	32	88	47	16	48	103	334
	(6.05 %)	(12.07 %)	(6.37 %)	(3.91 %)	(6.77 %)	(10.01 %)	(8.07 %)
Contribution to total number of PIC	9.58 %	26.35 %	14.07 %	4.79 %	14.37 %	30.84 %	100 %
No. of most parsimonious trees	1	6154	7520	10000	3300	9960	72
Tree Length	77	275	163	59	153	340	1102
CI	0.81	0.84	0.84	0.92	0.8	0.86	0.82
RI	0.87	0.87	0.85	0.91	0.86	0.88	0.85
Average number of changes per variable site (number of steps/number of variable sites)	1.35	1.35	1.3	1.23	1.43	1.31	1.38

### Maximum parsimony analyses

**Fig. 3:**
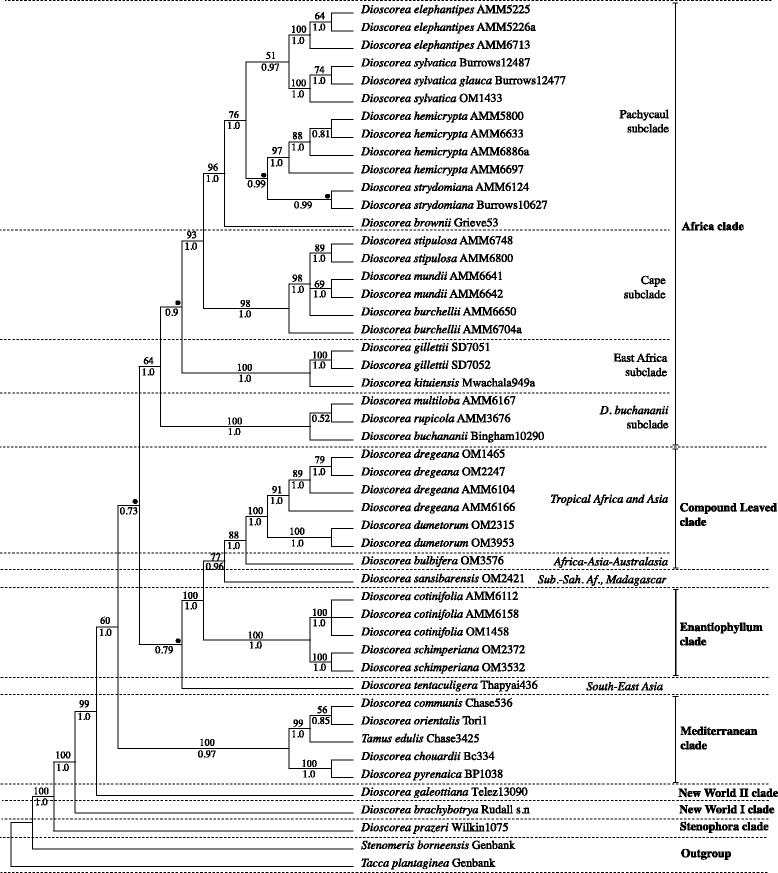
Bayesian 50 % MR consensus tree of African Dioscorea lineages with Bootstrap (BP) and Posterior Probabilities (PP) values located above and below branches, respectively. Branches that collapse in a polytomy in the MP strict consensus tree are illustrated by a •. “Clade” names (in bold on the figure) follow Viruel et al. [7]. Within the Africa clade, subclade names are proposed terminology used in this publication

MP analysis of each of the six regions resulted in trees that were similar in topology (Additional files 1, 2, 3, 4, 5 and 6), and were thus combined and treated as a single dataset. ILD test results provide support for congruence (*p* > 0.05). The *psaA-ycf3* region is significantly different from *rbcLa*, *matK*, *trnL-F*, *trnH-psbA*, and *rpl32-trnL* and probably caused by the *psaA-ycf3* sequence of *D. galeottiana*. However, the observed congruence between the trees obtained for each region separately and the ILD results (Table 3) support combining these regions. The statistics for the MP analysis for the combined data is presented in Table 2. From the heuristic search, we found 72 most parsimonious trees of which one is presented in the supplementary Additional file 7. The combined MP tree is largely congruent with that obtained from Bayesian analysis and therefore bootstrap values recovered in the MP analysis are plotted onto the Bayesian consensus tree (Fig. 3).

**Table 3:** Incongruence Length Difference (Farris test)﻿

	*rbcLa*	*matK*	*trnL-F*	*trnH-psbA*	*psaA-ycf3*	*rpl32-trnL*
*rbcLa*	-					
*matK*	0.362	-				
*trnL-F*	0.711	0.175	-			
*trnH-psbA*	0.679	**0.023**	0.474	-		
*psaA-ycf3*	**0.047**	**0.001**	**0.001**	0.757	-	
*rpl32-trnL*	0.922	0.750	0.748	0.549	**0.001**	-

Values in bold identify partitions significantly incongruent at *p*= 0.05

### Bayesian analysis

The Bayesian majority-rule consensus tree is presented in Fig. 3. Generally, the Bayesian analysis generated a better-supported topology than the MP analysis, resolving some polytomies observed in the MP results (see • in Additional file 7). *Dioscorea* is strongly supported as monophyletic (100 Bootstrap Percentage, BP; 1.0 Posterior Probabilities, PP). Within *Dioscorea* the topology is congruent with [6] and [7]. Three major clades are retrieved (Africa, Enantiophyllum/CL, Mediterranean), with the Central-American *D. galeottiana* (NWII clade), the Chilean *D. brachybotrya* (NWI clade) and the Asian *D. prazeri*, successively sister (99 BP/1.0 PP; 100 BP/1.0 PP; 100 BP/1.0 PP respectively) to these three core clades.

The Mediterranean clade is well-supported in both analyses (100 % BP; 0.97 PP), containing taxa from Spain and the south of France. Within this clade two well-supported lineages are identified, a “Spanish-Pyrenees mountain range” clade (100 BP/1.0 PP), and a more geographically dispersed taxa clade showing a wider distribution from Europe to the eastern Mediterranean and the Canary Islands (99BP/1.0 PP). The Mediterranean clade is weakly supported (0.73 PP) in the BI analysis as sister to a large clade comprising (1) the Enantiophyllum and the CL clades (including *Dioscorea sansibarensis*) and (2) the Africa clade. (1) comprises a combination of the weakly supported (BP < 50 %, 0.79 PP) *D. tentaculigera* sister to the Enantiophyllum clade and the CL clade, with *D. bulbifera* and *D. sansibarensis* successively sister to the CL clade. The large clade comprising (1) and (2) received no support in the MP analyses however it was weakly supported (0.73 PP) in BI.

The Africa clade is weakly supported in MP while strongly supported in the BI analyses (64 BP/1.0 PP); it includes the *D. buchananii*, East Africa, Cape and Pachycaul subclades. The Pachycaul subclade is strongly supported as monophyletic (96BP/1.0 PP) with *D. brownii* sister to all other pachycauls. *Dioscorea brownii* is a taxon restricted to montane grassland of KwaZulu-Natal (Fig. 2b and d) displaying a horizontal tuber a few centimetres in diameter with non-twining erect stems arising from vertical lobes (Fig. 1). Within the pachycaul group two sister lineages can be identified: 1) *D. hemicrypta* and *D. strydomiana* (0.99 PP). Both are characterised by a pachycaul tuber partially to wholly protruding above the substrate (Fig. 1), which reaches ca. 1 m in height and diameter in the latter. *Dioscorea hemicrypta* is endemic to the Little Karoo area South of the Swartberg Mountains in the Western Cape (Fig. 2b) while *D. strydomiana* has a single locality in Barberton area of Mpumalanga Province (Fig. 2), South Africa. 2) *D. elephantipes* and *D. sylvatica* have wide distribution ranges in South Africa (Fig. 2b), and are well supported as monophyletic in the BI analysis (0.97 PP) although it received weak support in the MP analyses (51 BP). These two taxa have well-developed pachycauls (Fig. 1), though that of *D. sylvatica* is usually below the substrate. The pachycaul of *D. elephantipes* can also reach ca. 1 m in height and diameter. Successively sister to the Pachycaul subclade are the Cape and the East Africa subclades (98 BP/1.0 PP and 100 BP/1.0 PP, respectively). The *D. buchananii* subclade of African *Dioscorea*, sister to the others, is resolved as the first branching lineage (100 BP/1.0 PP) within the Africa clade.

### Dating analysis

**Fig. 4:**
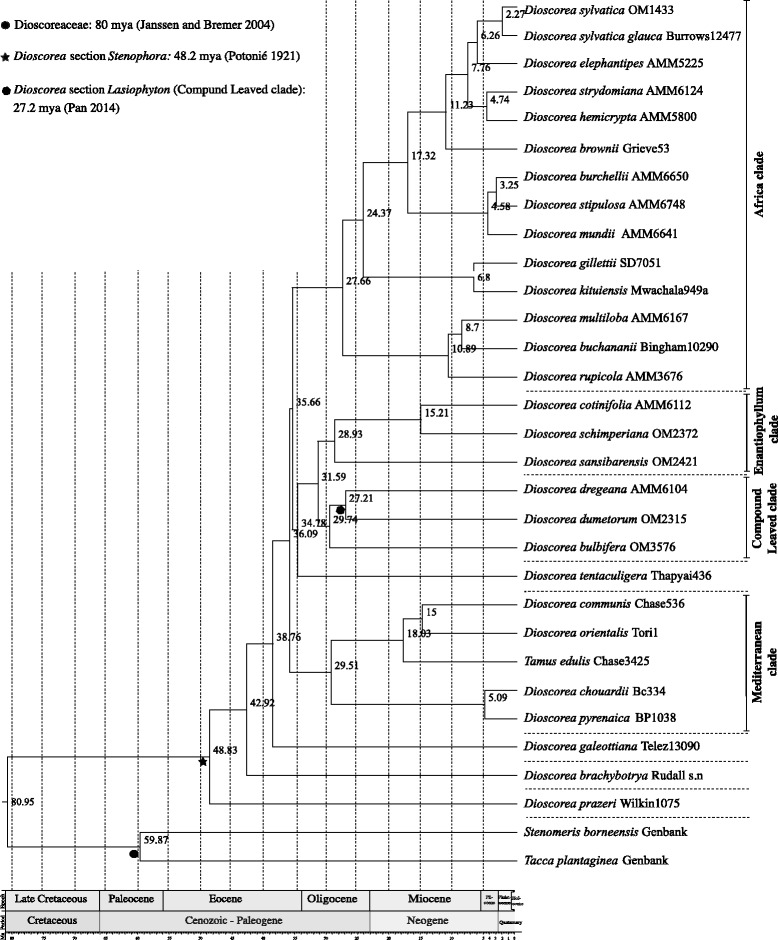
Beast chronogram of the African yam lineages dated using three calibrations points (● Dioscoreaceae: 80 mya [42], ★*Dioscorea* Stenophora clade crown node 48.2 mya [43] and  *Dioscorea dregeana* - *D. dumetorum* clade node in Counpound Leaved clade 27.2 mya [44]). Only the four major clades are displayed in the figure. For subclade information, refer to Fig. 3

The results of the dating analysis using BEAST are shown in Fig. 4. The topology retrieved is similar to that from BI. Results suggest an origin of the genus *Dioscorea* around 80.95 Ma and radiation from around 48.83 Ma. The first three diverging lineages of *Dioscorea*, the SE Asian *D. prazeri* (Stenophora clade) and the two New World taxa included in this study, *D. brachybotrya* (NWI) and *D. galeottiana* (NWII), split around 48.83 Ma, 42.92 Ma and 38.76 Ma respectively. Two successive splits at 36.09 Ma and 35.66 Ma were inferred for the ancestors of the Mediterranean clade and its sister lineage and for CL/Enantiophyllum and the Africa clade, respectively. The Mediterranean clade was estimated to have diversified at 29.51 Ma, and the ancestors of the South East-Asian *D. tentaculigera* and its sister group, the Enantiophyllum clade, and the CL clade at 34.78 Ma, 28.93 Ma and 29.74 Ma, respectively.

The successive splits of the Africa clade, the East Africa and sister lineage, and the core Cape and Pachycaul group were inferred to have occurred split at 27.66 Ma, 24.37 Ma and 17.32 Ma, respectively. Within the Pachycaul subclade a latter split of KwaZulu-Natal *D. brownii* and its sister lineage was dated at 11.23 Ma.

## Discussion

### Evolution of African yams

Data generated in this study produced a well-resolved dated phylogeny thus improving our understanding of the relationships within the Africa clade and more specifically within southern African *Dioscorea.* The current evolutionary study of yams focuses on southern African lineages, but representative taxa from other lineages were included to cover morphological and phylogenetic diversity of *Dioscorea*. The inferred phylogeny is congruent with previous studies (e.g. [6, 7]), though more largely sampled. Taxa occurring in southern Africa are nested within a strongly supported predominantly Old World clade (Mediterranean, Enantiophyllum, CL and Africa clades; Fig. 3 and 4), which likely originated in the Eocene. The Africa clade is further resolved into four subclades (Fig. 4) which are forest twiners with basally lobed leaves (*D. buchananii* subclade); savannah twiners (East Africa subclade); twiners in Cape forest and fynbos habitats (Cape subclade); and the diverse Pachycaul subclade comprising the open habitat elephant-foot yams with large, vertically oriented partially to wholly exposed tubers and stems with reduced to absent twining as well as a forest twiner (usually with similar but buried tubers) and an erect montane grassland taxon with a narrow horizontal tuber from which non-twining erect stems arise from vertical lobes.

Our analyses support that the Africa clade has four main subclades, (1) *D. buchananii* (2) East Africa, (3) Cape and (4) Pachycaul. Phylogenetic reconstruction placed the Cape subclade as sister to the Pachycaul and the Eastern African subclade sister to it. For the Pachycaul subclade, which was the main focus in this study, we found that *D. brownii* with a horizontal woody underground tuber from montane grassland in KwaZulu-Natal to be the earliest deriving taxon, sister to all four other pachycaul species (represented in two clades). The first include two taxa with restricted distribution (*D. hemicrypta* and *D. strydomiana*, respectively from the Little Karoo in the Western Cape and from a single locality in the Mpumalanga province) and displaying pachycauls located partially to completely above the substrate. The second group (*D. elephantipes* and *D. sylvatica*) has a much wider distribution. Both *D. elephantipes* and *D. strydomiana* possess pachycauls that can grow ca. 1 m in height and diameter.

Divergence estimation analyses, which were in broad agreement with those of Viruel et al. [7] suggest *Dioscorea* originated around 78 mya with a diversification around 48 mya. In the Old World clade, the Mediterranean taxa split from the African clade around 32.06 mya, with the latter diversifying around 26.74 mya. The East African taxa then diversify separately around 5.72 mya. The *D. buchananii* subclade diverges at 21.83 mya, and the split between the Cape subclade and the Pachycaul subclade is observed around 13.88 mya. These results confirm that the Africa clade forms part of a predominantly Old World clade, which originated during the Eocene. During the Oligocene the African continent was covered with dense and humid forest and characterized the period of development for thinner, underground perennial tuber and twining shoots displaying marginally winged (gliding) seeds, which favor their dispersal under canopy under low wind conditions. Through the Miocene climatic changes drove habitat opening with the appearance of grasslands in eastern Africa and Mediterranean climate in South African and in the Cape flora. Such changes, particularly the prominence of fire, influenced the development of an erect woody type of stem, below ground or partially to fully above ground, with corky bark as protection. Seed morphology also adapted to climatic and environmental conditions through the development of basally and apically winged seeds, which are more efficient when released at low height but needing higher wind speeds for efficient dispersal. In east Africa, seeds are wingless suggesting ants may be the mode of dispersal.

### Adaptation/colonization of yams to African biomes?

During the early Miocene, southern Africa was covered in forests [45–47], but increased edaphic heterogeneity due to uplifts [48] and increase in aridity and shifts in rainfall patterns after the formation of the Mediterranean climate resulted in the Cape flora (arid thickets, fire driven fynbos) and grasslands to the east [49]. The ancestor of the Africa clade would have thrived in open areas in forested habitats such as riverbanks as a twiner bearing perennial tubers. Colonization of non-forest habitats among Africa clade taxa involved shifts in stem and tuber morphology, most noticeable among the Pachycaul subclade, with large, long-lived tubers positioned fully or partially above sloping shale or rocky substrates (*D. elephantipes*, *D. hemicrypta*, *D. strydomiana*) versus fully below or at ground level [15] or sometimes fully exposed when occurring on/or between rocks (*D. sylvatica*). Erect, non-twining stems occur in taxa of frequently burned grasslands (*D. brownii*), or similarly burned open *Acacia* woodland with a strong grass understorey (*D. strydomiana*). The Pachycaul subclade taxa annually replace their photosynthetic tissues (stems and leaves) from the persistent tuber, a phenomenon observed in frequently burned habitats in southern Africa [50]. Only *D. brownii* and *D. strydomiana* occur within typical fire driven grassland habitats and their origin in late Miocene and Pliocene concurs with a similar time of origin of other southern African savannah flora [51]. Corky barks are observed covering the above-ground pachycauls both in fire prone grasslands and in (fire infrequent) thicket vegetation. Thick barks function to protect the plant from fire, an adaptation well documented in fire-prone areas [52–54], and may play an additional role of protection from herbivores. Damage to pachycauls, probably by porcupines, has been observed in populations of *D. hemicrypta* and *D. strydomiana*.

The Cape subclade comprises three species that occupy low elevation, high precipitation forested coastal habitats (*D. mundii*) or in middle to higher elevation fynbos heath vegetation (*D. burchellii*, *D. stipulosa*). All possess twining stems and have subterranean perennial tubers. Members of a clade occurring in both forest and fynbos habitats in the Cape flora is highly unusual, as adaptations for the forest environment (shade, no fire, richer soils) may not be advantageous in fynbos heath environment (open, frequent fires, nutrient poor). The habitats of *D. mundii* and *D. burchellii* are spatially separated by less than 10 km in the Eden district (that includes George and five surrounding municipalities) in the Western Cape. It has been noted that fynbos heath plants are more likely to disperse to similar environments occurring in distant lands (such as Australia) than to evolve adaptations to occupy a different (forest) biome nearby [55]. However, long distance dispersal is rare in *Dioscorea*, where wind dispersal is encountered almost without exception. The leaves of the two fynbos species are proportionally longer and narrower than those of *D. mundii*. We note that the Cape subclade is not sister to the Mediterranean subclade, the latter evolving independent traits observed in the Africa clade such as erect non-twining stems.

Within southern Africa *Dioscorea*, evolution into new non-forested biomes has occurred since the mid Miocene. The highest species diversity is in the east of the Cape (Fig. 2a; Eden, sensu Cowling & Pierce [56]), an area with complex geomorphology and climate, where several biomes (fynbos, forest, succulent karoo, thicket, grassland) are juxtapositioned. Speciation events accompanied evolution into the new biomes (e.g. grassland – *D. brownii*) or occurred subsequently in allopatry events (e.g. *D. strydomiana*/*D. hemicrypta*; *D. gillettii*/*D. kituiensis*; [10]).

**Fig. 5:**
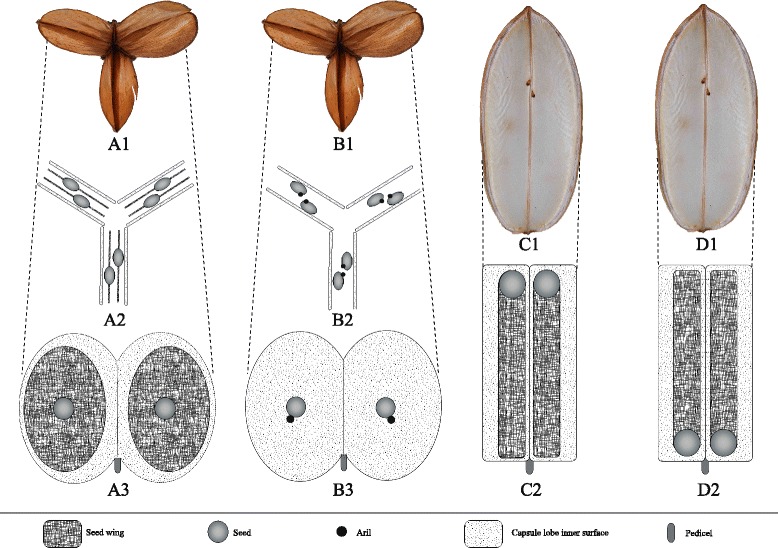
Capsular fruit forms with diagrammatic representations of seed wing shape, position and orientation within each capsule lobes in the Africa clade of *Dioscorea*. **a** Apical view of dehisced, empty capsule with rounded lobes (*A1*), diagrammatic cross-section of capsule through seeds and wings (*A2*) and diagrammatic side view of two opened lobes containing seeds winged all around the margin (*A3*): *D. buchananii* subclade. **b** Same views of similar capsule (*B1*) containing wingless, arillate seeds (*B2*, *B3*): East Africa subclade; **c** Side view of two opened oblong capsular lobes without seeds (*C1*) and diagrammatic side view of basally winged seeds (*C2*) associated with this capsule lobe shape: Cape subclade; **d** Same views of similar capsule lobes containing apically winged seeds (*D1*, *D2*); Pachycaul subclade

The opening of vegetation during the Miocene in southern Africa had an important influence on seed morphology and therefore on their dispersal mode. In forest environments where yam species grow below the forest canopy and generally have a twining habit, lens-shaped seeds are characterized by flat papery wings all round the margin (Fig. 5A2 and A3), which allow them to glide effectively, even with low wind speeds. This is observed in all species of the *D. buchananii* subclade. According to Burkill [57] this is the optimal form for dispersal when seeds are released from greater height and in light winds, the conditions that pertain to forest climbers under a canopy. The two species of the East African subclade both possess wingless seeds but an aril (or elaiosome; Fig. 5B2 and B3) is present suggesting that myrmecochory may be its mode of dispersal [16]. However it remains confusing why such a trait evolved in habitats dominated by *Acacia*-*Commiphora* and *Terminalia*-*Combretum,* open savannah woodlands where wind dispersal is widespread, and where ant dispersal may not be dominant [58]. Contrarily, the two fynbos species are wind dispersed even though ant dispersal is thought to be prevalent in that habitat [59]. *Dioscorea burchellii* in particular is low growing and often concealed among fynbos shrubs. Loss of seed wings has arisen independently in the Mediterranean taxa *D. pyrenaica* and *D. chouardii* as well as in New World taxa such as *D. sphaeroidea* R. Couto & J.M.A. Braga, *D. biloba* (Phil.) Caddick & Wilkin and *D. humilis* Colla. It is likely to be linked to switches in dispersal mode. The Cape and Pachycaul subclade taxa have clearly evolved independently but display similar functional features, respectively basally and apically papery winged lens-shaped seeds (Fig. 5C2 and D2). Both of these seed wing traits allow the seeds to spin in flight in a similar manner to a samaroid fruit. It is likely that that basally winged seeds are easier to dislodge than apically winged seeds but both subclades still share convergent dispersal methods. Basally and apically winged seeds are features that have evolved on many occasions and have been observed in groups that generally produce fruits close to ground level. According to Burkill [57] such features are particularly efficient in open habitat where wind speeds are higher.

Interestingly, the only southern African species of the pan-palaeotropical Enantiophyllum clade, *D. cotinifolia*, differs from all other member of that clade by possessing an apically winged seed like that illustrated in Fig. 5D2; the rest have marginally winged (gliding) seeds (like those in Fig. 5A2 and 5A3). In South Africa, this taxon tends to occur in open seasonal woody vegetation (e.g.) that is less dense than the vegetation inhabited by tropical species, and its radiation at the end of the Miocene at similar time as South African taxa suggest parallel evolution in similar type of open environment.

Overall, apart from seed wing form, reproductive morphology in the Africa clade has been less impacted by biome shifts than vegetative morphology. The only significant variation in floral form is found in *D. rupicola*, which has only 3 stamens and a discoid torus (as opposed to 6 and a thin, bowl-shaped torus); it also has narrower tepals than those in *D. buchananii* or *D. multiloba* and the flower is held pendent. However, these changes are probably linked to the shift to a different pollinator within the forest biome in which this species, principally found the Drakensberg and high elevations in the Eastern Cape [8].

## Conclusions

Diversification out of forest is associated with a major increase in perennial tuber size and change in tuber orientation from horizontal to vertical, both of which presumably underlie the development of pachycauly. There is also a shift in stem habit, from twining on supporting vegetation to erect and self-supporting. This diversification does not show association with reproductive morphology, except in the seed wing, which has switched from being winged all round the seed margin (to promote gliding flight) to only on its basal or apical side (generating spinning flight). The wing has even been completely replaced by an elaiosome in two species. The single pollinator shift event is observed within the forest biome.

Although only *D. brownii* and *D. strydomiana* currently occur within typical fire driven grassland, the transition of the vegetation from closed habitat to savanna grasslands occurring during the Miocene and Pliocene, with an associated increase in fire regime and similar time of origin of other southern African savannah flora elements, suggests that this change has influenced the development of corky barks covering the above-ground pachycauls and therefore the origin of efficient fire and perhaps herbivory protection.

## Additional files

Additional file 1:
*rbcLa* MP tree. One of the most equally parsimonious tree generated from the Maximum Parsimony (MP) analysis using *rbcLa* sequence dataset. Values above branches are number of steps and values below branches are reported percentage of Bootstrap support values. Collapsing branches from the strict consensus tree obtained in the combined Maximum Parsimony (MP) analysis are illustrated with a •. (PDF 210 kb)
Additional file 2:
*matK* MP tree. One of the most equally parsimonious tree generated from the Maximum Parsimony (MP) analysis using *matK* sequence dataset. Values above branches are number of steps and values below branches are reported percentage of Bootstrap support values. Collapsing branches from the strict consensus tree obtained in the combined Maximum Parsimony (MP) analysis are illustrated with a •. (PDF 230 kb)
Additional file 3:
*trnL-F* MP tree. One of the most equally parsimonious trees generated from the Maximum Parsimony (MP) analysis using *trnL-F* sequence dataset. Values above branches are number of steps and values below branches are reported percentage of Bootstrap support values. Collapsing branches from the strict consensus tree obtained in the combined Maximum Parsimony (MP) analysis are illustrated with a •. (PDF 221 kb)
Additional file 4:
*trnH-psbA* MP tree. One of the most equally parsimonious trees generated from the Maximum Parsimony (MP) analysis using *trnH-psbA* sequence dataset. Values above branches are number of steps and values below branches are reported percentage of Bootstrap support values. Collapsing branches from the strict consensus tree obtained in the combined Maximum Parsimony (MP) analysis are illustrated with a •. (PDF 206 kb)
Additional file 5:
*psaA-ycf3* MP tree. One of the most equally parsimonious trees generated from the Maximum Parsimony (MP) analysis using *psaA-ycf3* sequence dataset. Values above branches are number of steps and values below branches are reported percentage of Bootstrap support values. Collapsing branches from the strict consensus tree obtained in the combined Maximum Parsimony (MP) analysis are illustrated with a •. (PDF 220 kb)
Additional file 6:
*rpl32-trnL* MP tree. One of the most equally parsimonious trees generated from the Maximum Parsimony (MP) analysis using *rpl32-trnL* sequence dataset. Values above branches are number of steps and values below branches are reported percentage of Bootstrap support values. Collapsing branches from the strict consensus tree obtained in the combined Maximum Parsimony (MP) analysis are illustrated with a •. (PDF 224 kb)
Additional file 7: Combined MP tree. One of the most equally parsimonious trees generated from the Maximum Parsimony (MP) analysis using *the combined* sequence dataset. Values above branches are number of steps and values below branches are reported percentage of Bootstrap support values. Collapsing branches from the strict consensus tree obtained in the combined Maximum Parsimony (MP) analysis are illustrated with a •. (PDF 264 kb)

## Abbreviations

CL: Compound-Leaved
CTAB: Hexadecyltrimethylammonium bromide
ILD: Incongruence Length Difference
NWI: New World I (clade)
NWII: New World II (clade)
*Xdh* (nuclear region *Xdh*): Newly Sequenced Nuclear Gene
