# Supplementary figures and images for: Diversification into novel habitats in the Africa clade of Dioscorea (Dioscoreaceae): erect habit and elephant’s foot tubers
Source: BMC Evol Biol. 2016 Nov 8;16:238. doi: 10.1186/s12862-016-0812-z (PMC5100304; doi:10.1186/s12862-016-0812-z)

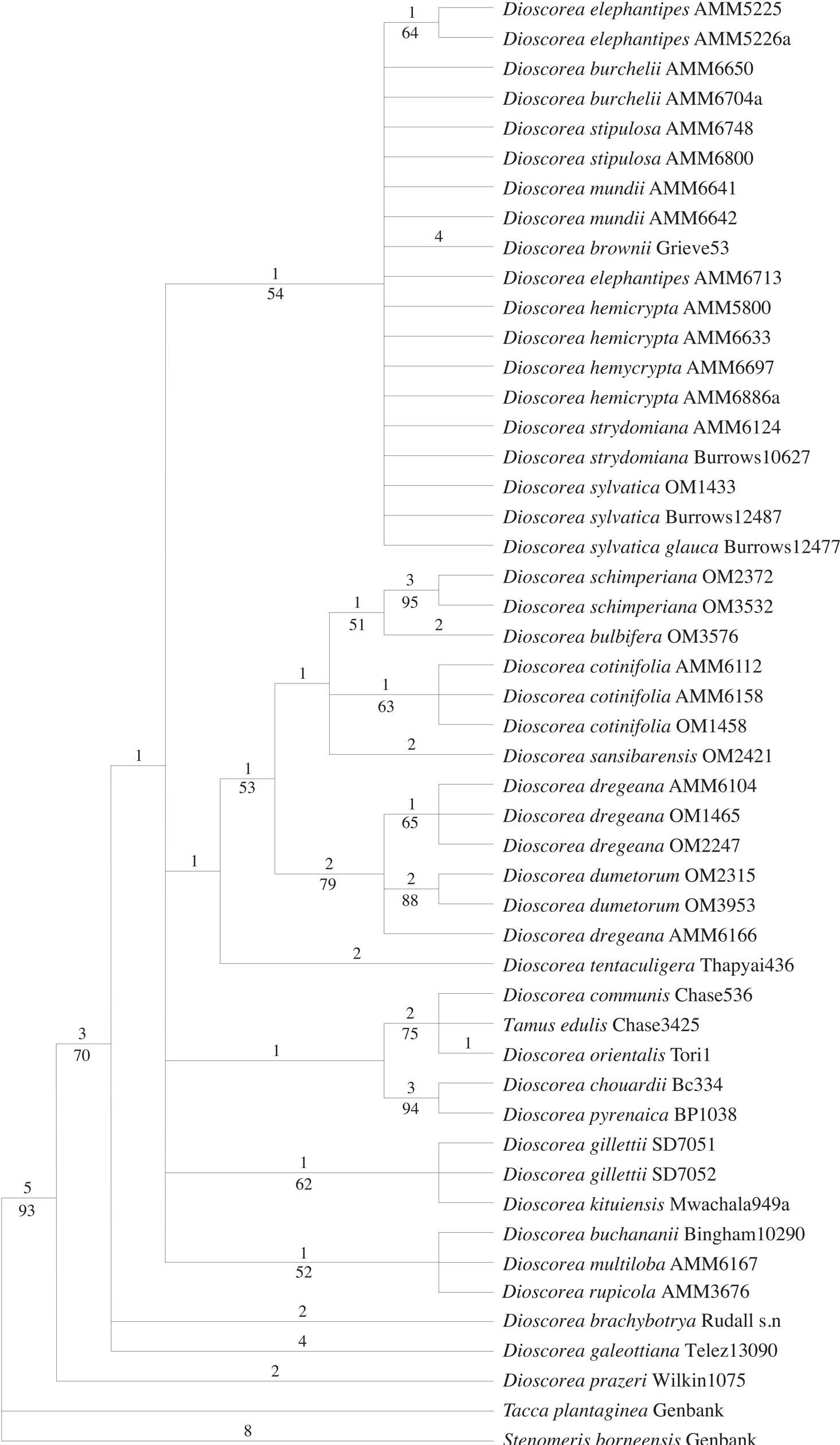

Supplement: Additional file 1: — rbcLa MP tree. One of the most equally parsimonious tree generated from the Maximum Parsimony (MP) analysis using rbcLa sequence dataset. Values above branches are number of steps and values below branches are reported percentage of Bootstrap support values. Collapsing branches from the strict consensus tree obtained in the combined Maximum Parsimony (MP) analysis are illustrated with a •. (PDF 210 kb) [file 12862_2016_812_MOESM1_ESM.pdf]

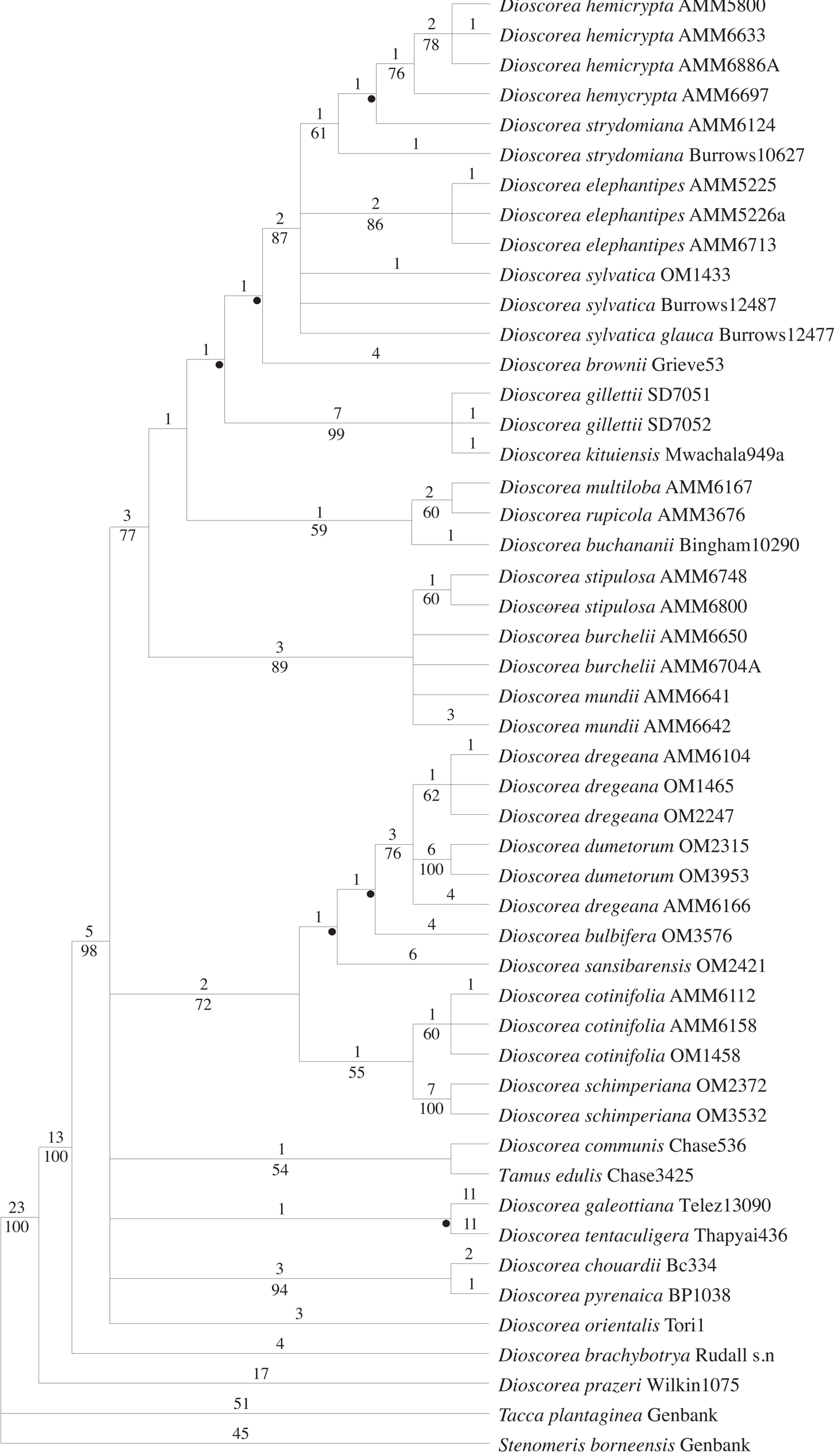

Supplement: Additional file 2: — matK MP tree. One of the most equally parsimonious tree generated from the Maximum Parsimony (MP) analysis using matK sequence dataset. Values above branches are number of steps and values below branches are reported percentage of Bootstrap support values. Collapsing branches from the strict consensus tree obtained in the combined Maximum Parsimony (MP) analysis are illustrated with a •. (PDF 230 kb) [file 12862_2016_812_MOESM2_ESM.pdf]

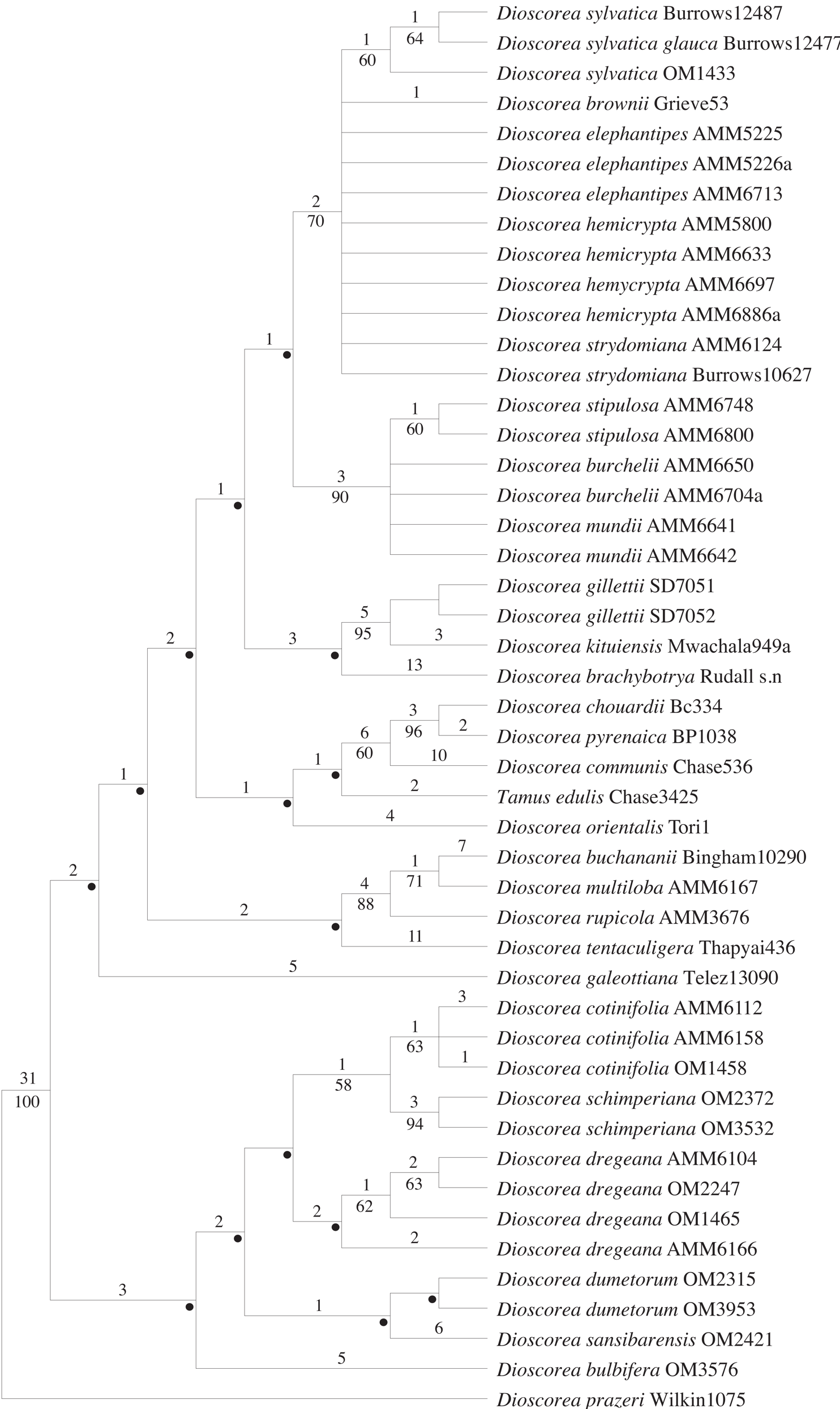

Supplement: Additional file 3: — trnL-F MP tree. One of the most equally parsimonious trees generated from the Maximum Parsimony (MP) analysis using trnL-F sequence dataset. Values above branches are number of steps and values below branches are reported percentage of Bootstrap support values. Collapsing branches from the strict consensus tree obtained in the combined Maximum Parsimony (MP) analysis are illustrated with a •. (PDF 221 kb) [file 12862_2016_812_MOESM3_ESM.pdf]

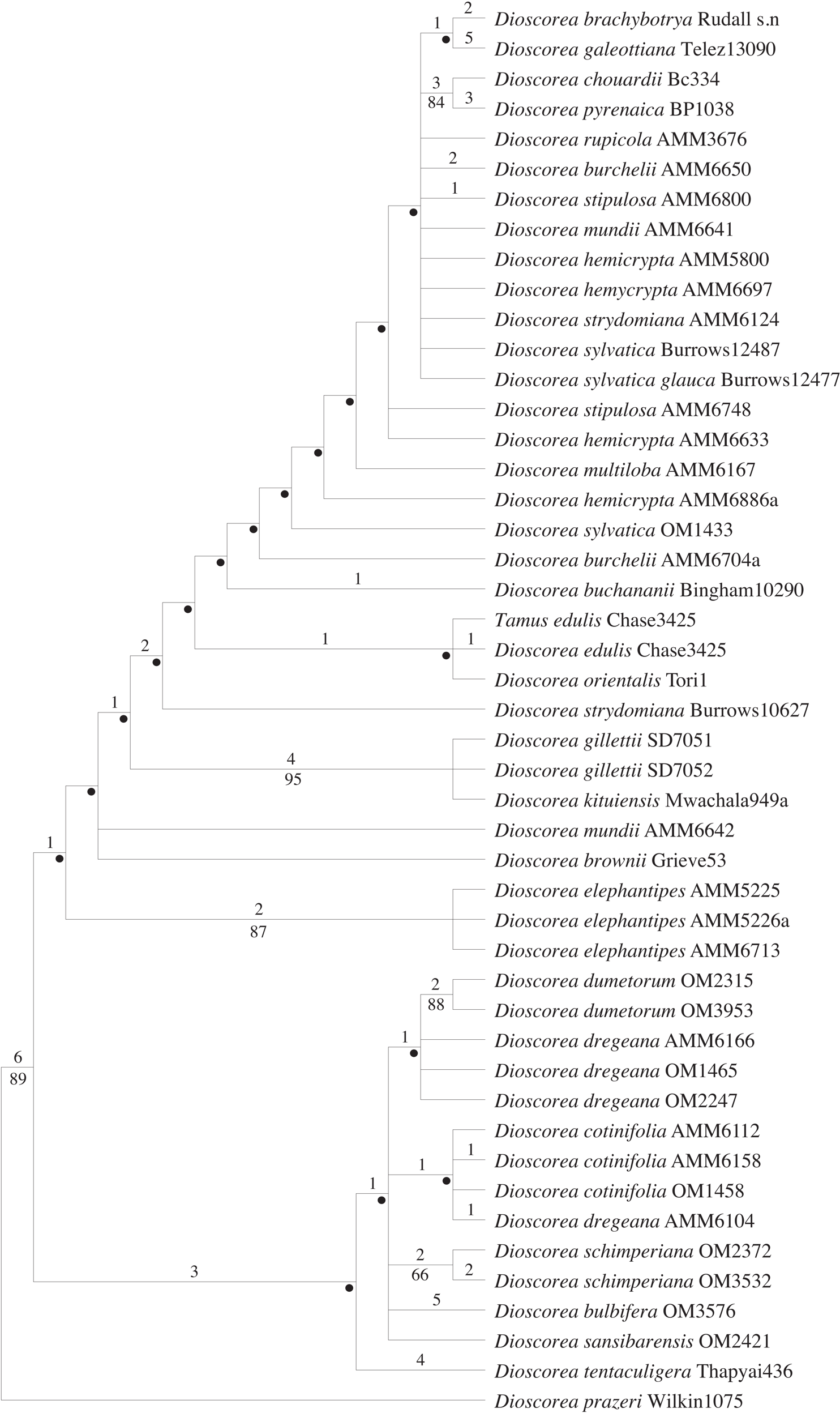

Supplement: Additional file 4: — trnH-psbA MP tree. One of the most equally parsimonious trees generated from the Maximum Parsimony (MP) analysis using trnH-psbA sequence dataset. Values above branches are number of steps and values below branches are reported percentage of Bootstrap support values. Collapsing branches from the strict consensus tree obtained in the combined Maximum Parsimony (MP) analysis are illustrated with a •. (PDF 206 kb) [file 12862_2016_812_MOESM4_ESM.pdf]

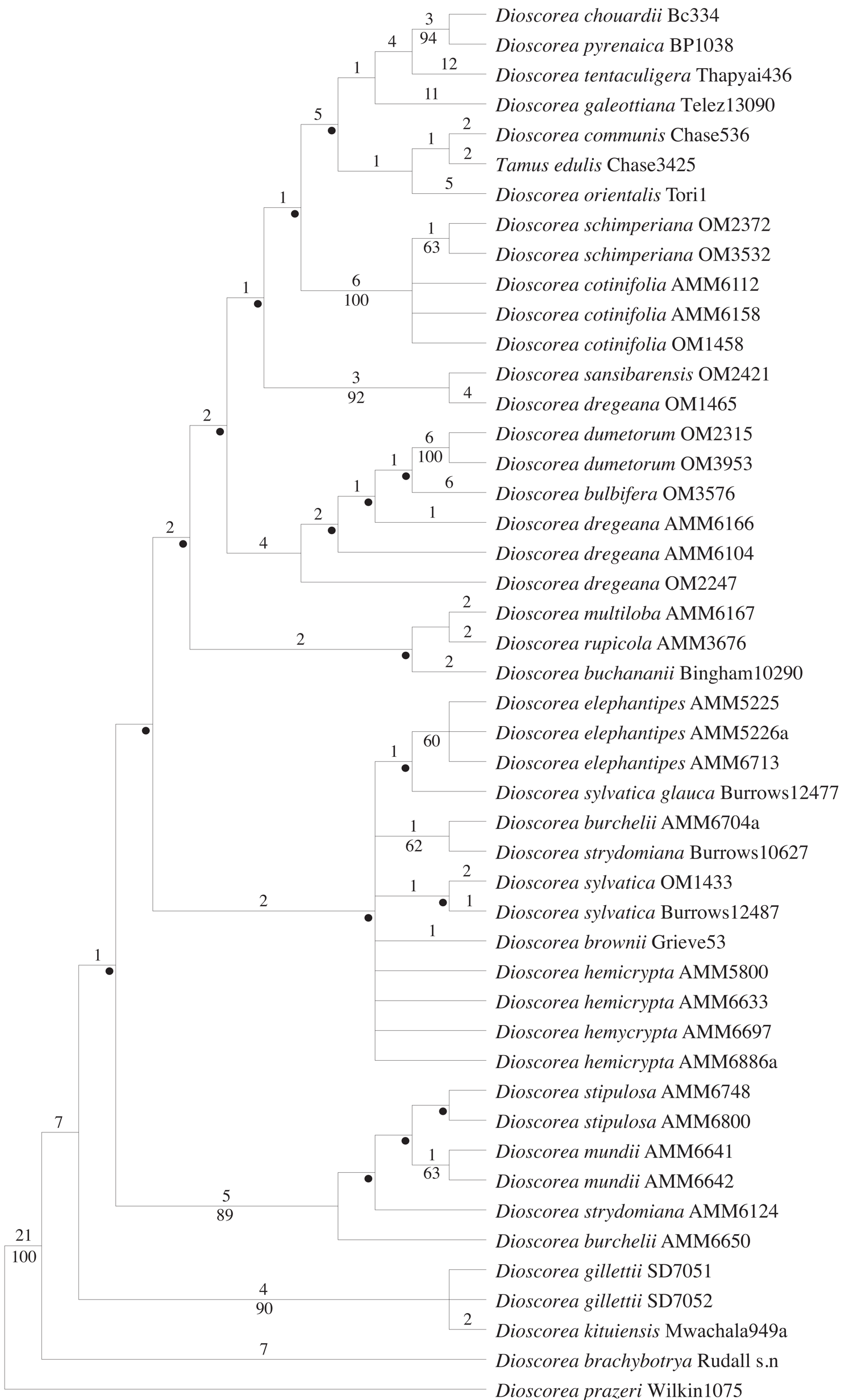

Supplement: Additional file 5: — psaA-ycf3 MP tree. One of the most equally parsimonious trees generated from the Maximum Parsimony (MP) analysis using psaA-ycf3 sequence dataset. Values above branches are number of steps and values below branches are reported percentage of Bootstrap support values. Collapsing branches from the strict consensus tree obtained in the combined Maximum Parsimony (MP) analysis are illustrated with a •. (PDF 220 kb) [file 12862_2016_812_MOESM5_ESM.pdf]

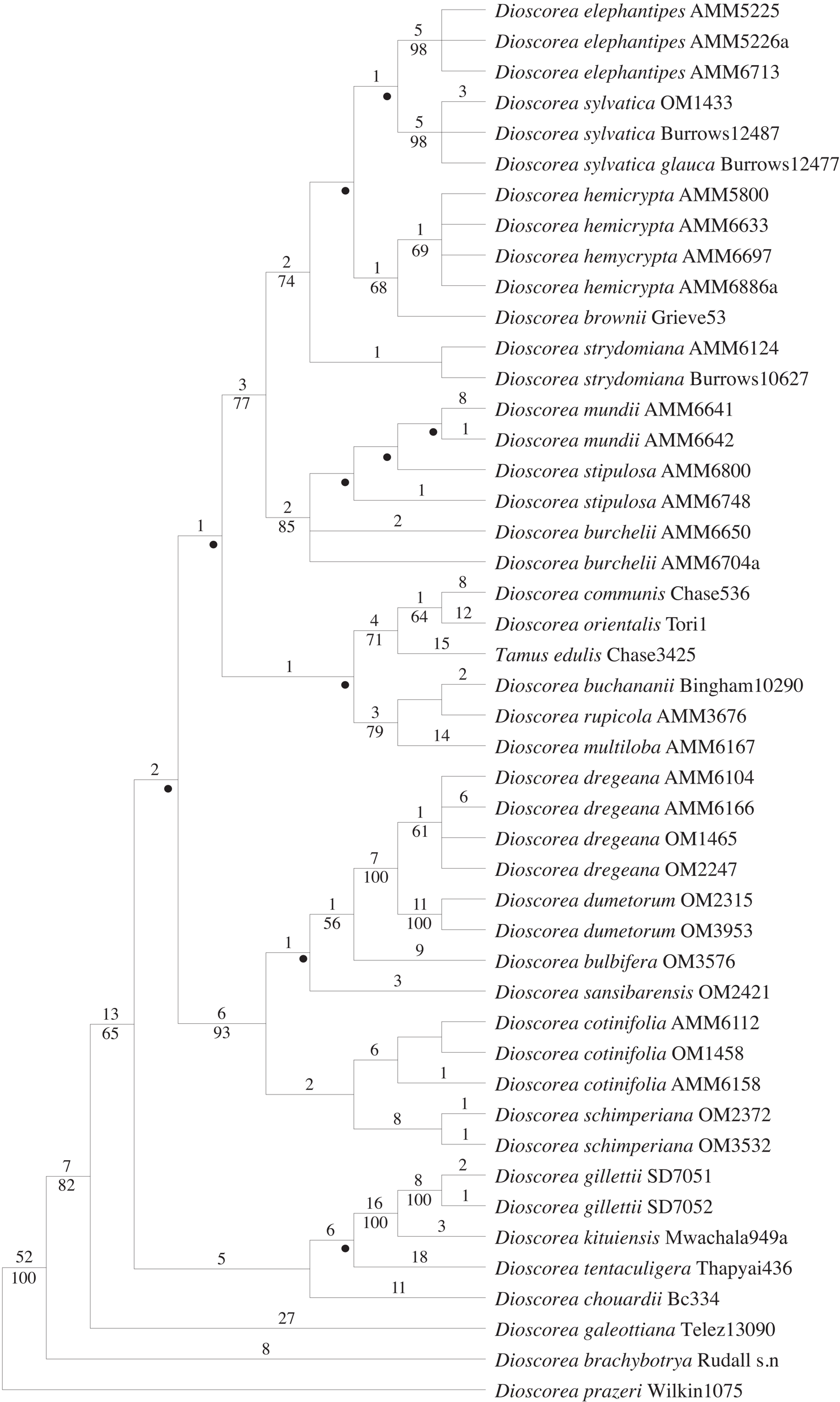

Supplement: Additional file 6: — rpl32-trnL MP tree. One of the most equally parsimonious trees generated from the Maximum Parsimony (MP) analysis using rpl32-trnL sequence dataset. Values above branches are number of steps and values below branches are reported percentage of Bootstrap support values. Collapsing branches from the strict consensus tree obtained in the combined Maximum Parsimony (MP) analysis are illustrated with a •. (PDF 224 kb) [file 12862_2016_812_MOESM6_ESM.pdf]

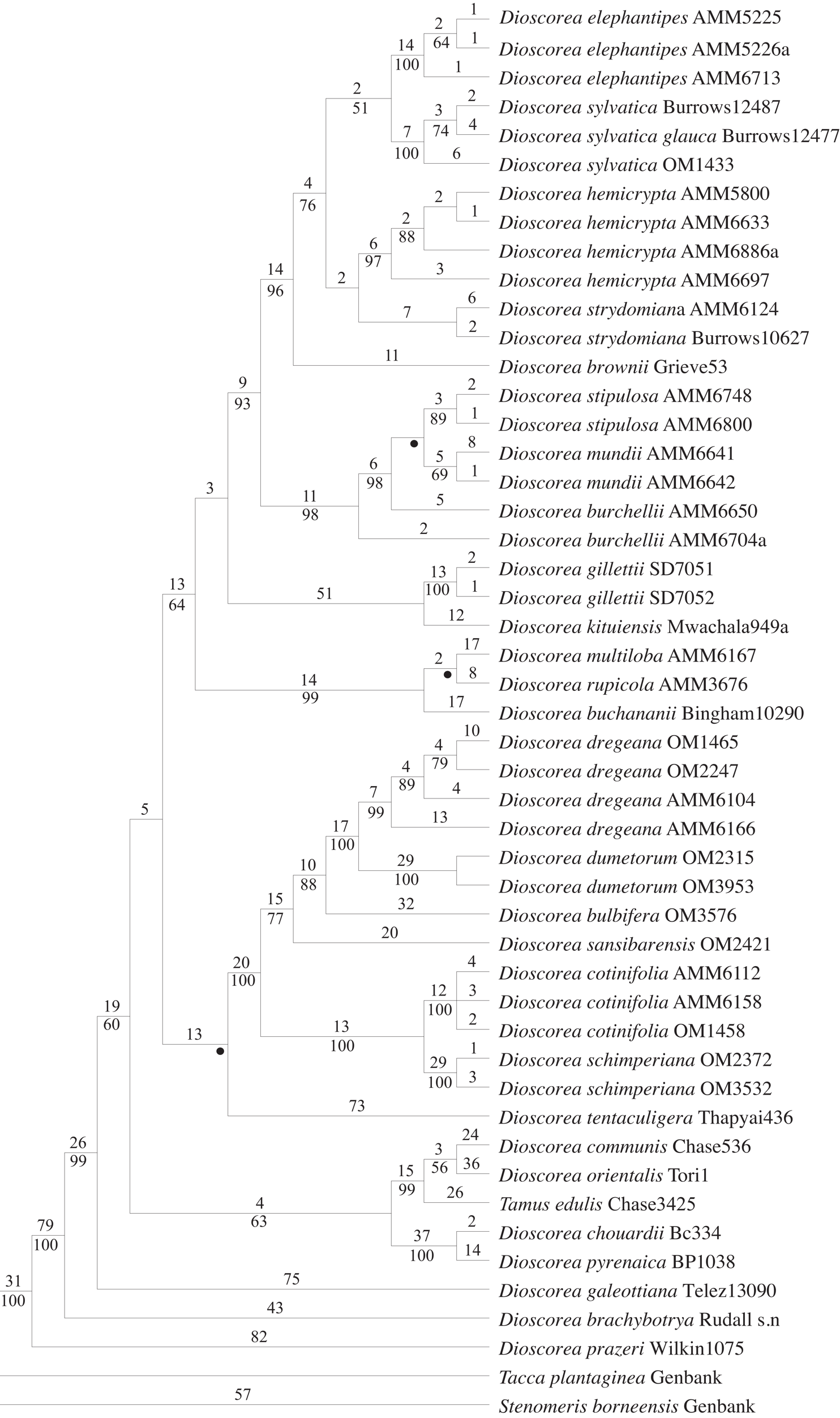

Supplement: Additional file 7: — Combined MP tree. One of the most equally parsimonious trees generated from the Maximum Parsimony (MP) analysis using the combined sequence dataset. Values above branches are number of steps and values below branches are reported percentage of Bootstrap support values. Collapsing branches from the strict consensus tree obtained in the combined Maximum Parsimony (MP) analysis are illustrated with a •. (PDF 264 kb) [file 12862_2016_812_MOESM7_ESM.pdf]
